# Pepper Crop Improvement Against Cucumber Mosaic Virus (CMV): A Review

**DOI:** 10.3389/fpls.2020.598798

**Published:** 2020-12-10

**Authors:** Ning Li, Chuying Yu, Yanxu Yin, Shenghua Gao, Fei Wang, Chunhai Jiao, Minghua Yao

**Affiliations:** Hubei Key Laboratory of Vegetable Germplasm Innovation and Genetic Improvement, Cash Crops Research Institute, Hubei Academy of Agricultural Sciences, Wuhan, China

**Keywords:** *Capsicum*, cucumber mosaic virus, resistance, marker-assisted breeding, quantitative trait loci

## Abstract

Cucumber mosaic virus (CMV) is a prevalent virus affecting the quality and yield of pepper, resulting in yield losses of greater than 80% during severe local epidemics. Cultural practices and the heavy use of agrochemicals are the most common control measures for CMV. Sources of resistance provide a practical reference and a basis for breeding for CMV resistance. Genetic factors underlying CMV resistance have been studied and advanced breeding lines and cultivars with improved resistance have been developed by traditional breeding methods. Additionally, QTLs or genes for CMV resistance have been identified and can be utilized for marker-assisted resistance breeding. This review focuses on status and prospect of CMV against different virus strains, host resistance, and its applied genetics. With the advent of novel technologies, more useful markers and precise approaches can facilitate the progress for improving CMV resistance in *Capsicum*.

## Introduction

Pepper (*Capsicum* spp.) is an important vegetable and spice crop worldwide owing to its color, taste, pungency, flavor, and aroma (Rohini and Lakshmanan, 2017). In 2018, approximately 36.7 million tons of fresh pepper fruit and 4.16 million tons of dried pods were harvested from 3.76 million hectares worldwide (FAOSTAT, 2018)^1^. Among vegetable crops in China, pepper has the largest cultivated area, with an annual planting area of 2.13 million hectares and output of more than 25 million tons (Wang et al., 2019). Pepper production is frequently threatened by many biotic factors, such as diseases, weeds, and pests, including bacterial wilt, phytophthora root rot, cucumber mosaic virus (CMV), root-knot nematodes, aphids, and thrips (Ridzuan et al., 2018). Among these threats, CMV is the most significant constraint to pepper production and it has been found in most of pepper cultivating countries cross the worldwide (Doolittle, 1916; Jagger, 1916; Palukaitis et al., 1992). CMV is a seed-borne disease in pepper resulting in yield losses of over 80% in years of severe local epidemics (Joshi and Dubey, 1973; Florini and Zitter, 1987; Green and Kim, 1991; Avilla et al., 1997). In China, CMV results in marketable yield losses of 20 to 40% in most of the pepper-growing regions (Wu et al., 2006; Zhao et al., 2009). The host range of CMV is very broad, affecting more than 1200 plant species belonging to 100 families, including other important solanaceous crops (e.g., potato, tobacco, and tomato) (Watterson, 1993; Jacquemond, 2012). Additionally, over 80 species of aphids are known to transmit the virus in a non-persistent manner (Palukaitis and Garcíaarenal, 2003).

**FIGURE 1:**
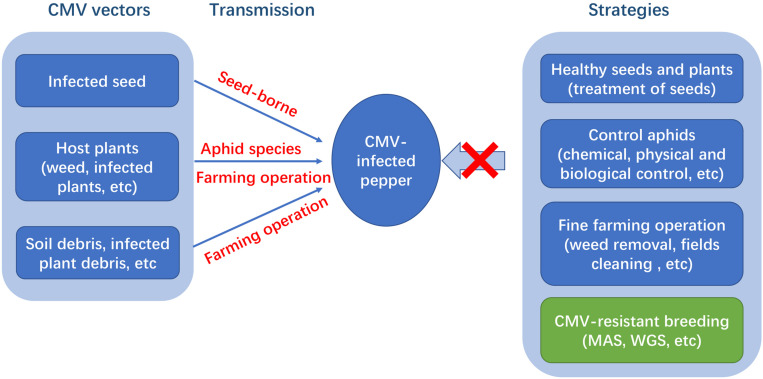
CMV vectors, virus transmission and strategies for managing CMV in pepper.

Several strategies have been implemented to control CMV, including cultural measures (e.g., weed removal, the eradication of infected plants, and disease-free seed use) and chemical and biological pesticides, such as pyrethroids, abamectin, and carbamate, to control aphids (insect vectors) (Figure 1). The broad host range and large number of insect vectors make disease control very difficult (Palukaitis et al., 1992). Additionally, chemical insecticides are considered uneconomical and environmentally unfriendly. Thus, the most effective, sustainable, and long-lasting strategy for the control of CMV is the development of disease-resistant pepper cultivars or hybrids (Yao et al., 2013). Some sources of resistance to CMV in *Capsicum* spp. have been identified and utilized for breeding (Green and Kim, 1994). However, the levels of CMV resistance in commercial lines are still insufficient (Pochard and Daubèze, 1989). Hence, it is of primary importance to screen new sources of CMV resistance. Additionally, the genetic basis of resistance to CMV in *Capsicum* is complex, unclear, and even controversial in some cases. Most studies have found partial resistance mediated by multiple loci, and a few studies reported dominance or recessive monogenic. Therefore, it requires the clarification the genetic basis of resistance as well as resistance mechanisms and the development of molecular markers linked to resistance loci. This information will contribute to breeding for resistant pepper cultivars. The current study provides an overview of CMV diseases in pepper, focusing on the characteristics of virus strains, mechanisms underlying host resistance, genetics, and efforts to breed pepper lines and cultivars with improved resistance.

## Disease Symptoms and Management

Cucumber mosaic virus was first discovered in cucumbers and muskmelons in Michigan, United States and in cucumbers in New York, United States in 1916 (Doolittle, 1916). It is now known to occur worldwide in both temperate and tropical climates, affecting many agricultural and horticultural crops, as one of the most prevalent viruses. Common symptoms of CMV infection in peppers are mottle, mosaic, vein clearing, yellow discoloration, narrowing, or shoe-stringing (Conti et al., 1979; Green and Kim, 1991; Zitter and Murphy, 2009). The symptoms of CMV infection in peppers are highly dependent on plant age; young plants tend to show more severe symptoms, while plants at later stages of development may be asymptomatic (Garcia-Ruiz and Murphy, 2011; Kenyon et al., 2014). When plants are young at the time of infection, early leaves are slightly wrinkled or bumpy and pale green. During growth, the foliage may develop oak-leaf patterns or ringspot. As new leaves emerge, they develop a chlorotic mosaic pattern that tends to encompass the entire leaf (Zitter and Murphy, 2009). CMV-infected pepper plants also tend to show severe stunting and reduced flower formation and set. The fruits are small, malformed, bumpy, patchily discolored, and may show depressed spots or necrotic lesions, leading to significantly reduced fruit yield and quality (Sugiura et al., 1975; Lockhart and Fischer, 1976; Green and Kim, 1994; Zhao et al., 2009). Furthermore, CMV composite infections with other viruses are commonly observed in the field, and the symptoms are usually difficult to distinguish from those of single CMV infection. Several studies have also reported severe synergy in some CMV strains coinfected other viruses (e.g., CMV and PepMov) in pepper plants (Tien et al., 1987; Palukaitis et al., 1992; Zhao et al., 2004; Gao et al., 2016).

Cucumber mosaic virus is transmitted by the mechanical inoculation of plant sap and aphids are the most important means of natural transmission (Edwardson and Christie, 1986). More than 80 aphid species have been described as CMV vectors and transmit the virus in a non-persistent, stylet-borne manner. CMV does not reproduce in its aphid vector and is not transmitted to progeny aphids. For vegetables, *Myzus persicae* and *Aphis gossypii* are the most efficient vectors for virus transmission (Conti et al., 1979). A major characteristic of non-persistent transmission is that aphids acquire the virus for short periods of time (normally a few seconds) and lose the virus after normal feeding on plants. CMV can also be transmitted via infected seed, infected crop debris, non-vectored soil debris, pollen, and other routes (Pares and Gunn, 1989; Zhou et al., 1994; Chen et al., 2000).

The main biological strategies for managing CMV is the eradication of infected plants from field plot. CMV has an extremely wide alternative host range, including many species of weeds, such as *Carex vulpine, Solanum nigru*, and *Datura stramonium* (Kazinczi et al., 2004; Juan et al., 2006). These weed hosts in nearby field plots are important potential virus sources for aphid transmission to pepper. When pepper crops are absent, some weed species are perennial depositories for non-persistently transmitted viruses by viruliferous vectors. Additionally, the virus can remain viable in plant debris in the soil for several months. Thus, CMV infection can occur from infected soil debris via non-vectored soil transmission (Pares and Gunn, 1989). Second, owing to the seed-borne nature, the use of healthy seeds and plants for production is necessary. The control of CMV should start by only using clean and disease-free seeds. The treatment of seeds with 15% trisodium phosphate solution is an effective method to reduce the incidence of viral disease (Rast and Stijger, 1987). Third, the application of chemical insecticides and biological pesticides to control insect vectors can be effective. However, it is not a sustainable strategy, since populations of beneficial insects (such as coccinellidae) may be adversely affected by insecticides (Wang and Uchida, 2014). Moreover, the use of insecticides can result in the rapid development of insecticide-resistant populations. High costs and environmental consequences are additional issues. Thus, the development of disease-resistant pepper cultivars is an important approach to overcoming the threat of CMV.

## Overview of the Virus

### Viral Structure

**FIGURE 2:**
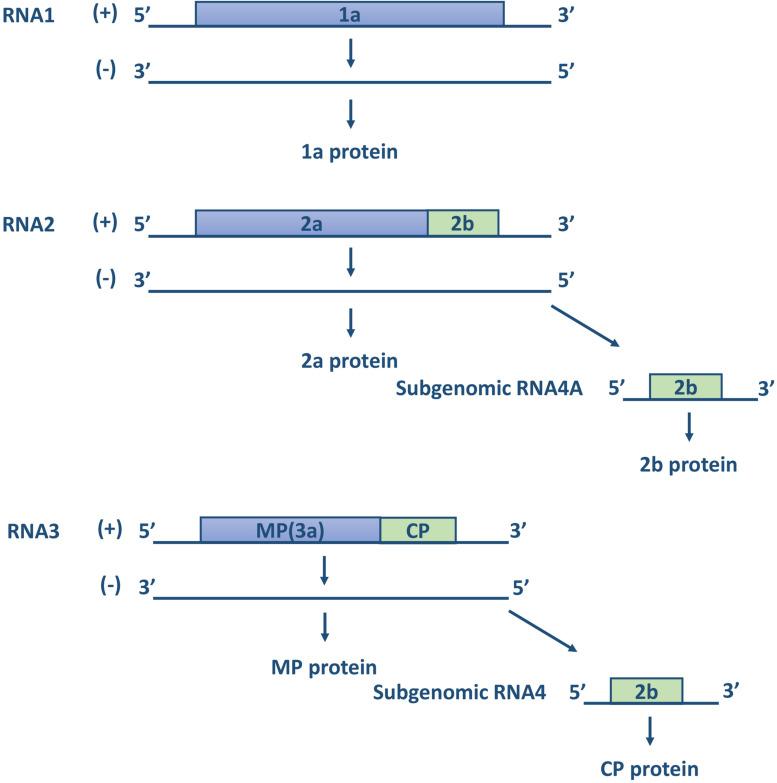
The structure of genomic RNAs of CMV. The genomic plus-strand and minus-strands are indicated with a “+” or “–” (modified according to Zitter and Murphy, 2009).

Cucumber mosaic virus is the type member of the genus *Cucumovirus* in the family *Bromoviridae* and the superfamily of alpha-like viruses (Goldbach, 1987; Kenyon et al., 2014). It is a multicomponent virus with a single-stranded positive-sense RNA genome, consisting of three genomic RNAs, designated RNA1, RNA2, and RNA3, and subgenomic RNA4 and RNA4A (Sivakumaran et al., 2000, 2002; Figure 2). RNA1 encodes a single protein, referred to as 1a, which is related to the replication of the viral genome. The sequence of protein 1a is highly conserved, with two functional domains. The N-terminal domain has methyltransferase activity for the addition of a cap structure to the 5′-terminus of genomic and subgenomic RNAs, while the C-terminal domain of the 1a protein is a putative helicase, which functions to “unwind” the double-stranded RNA that develops during viral replication (Zitter and Murphy, 2009). RNA2 encodes two proteins, referred to as 2a and 2b. The 2a protein has RNA-dependent RNA polymerase (RdRp) activity and participates in the viral replication process together with the 1a protein. The 2b protein is translated from a separate (subgenomic) RNA strand, referred to as RNA4A, within the 3′ portion of RNA2. The CMV 2b protein is involved in long-distance virus movement, expression of systemic symptoms, and the suppression of gene silencing (Ding et al., 1994; Brigneti et al., 1998). The CMV 2b protein inhibits the plant initiation of gene silencing in distant tissues, thereby allowing CMV to invade plant tissues (Zitter and Murphy, 2009). RNA3 encodes the 3a protein and the coat protein (CP) expressed from subgenomic RNA4. The 3a protein is associated with intercellular movement and long-distance transport of the virus in the host; accordingly, it is also called movement protein (MP). CP is the only protein associated with virus particles and is the sole determinant of transmission by aphid vectors. Minor changes in the sequence of the viral coat protein can affect the ability of CMV strains to be transmitted by aphids (Palukaitis et al., 1992). Moreover, according to Mochizuki and Ohki (2005), certain amino acid positions of CP have important functions, such as residue 129, whose mutation affect host symptoms and the transmission efficiency of the virus (Table 1).

**TABLE 1:** The function of protein encoded by CMV RNAs.

RNAs	Proteins	Function
RNA1	1a	Replication of the viral genome
RNA2	2a	RdRp activity and participates in the viral replication process
Subgenomic RNA4A	2b	Influence the systemic infection of the virus and inhibit virus-mediated gene silencing
RNA3	MP (3a)	Virus cell-to-cell movement, intraplant long distance movement
Subgenomic RNA4	CP	The sole determinant for transmission by aphid vectors

### CMV Strains

The traditional classification of CMV strains was based on pathotype and morphological symptoms in different host species (Kaper and Waterworth, 1981). For example, six different CMV isolates were identified by observed varying symptoms on host plants (Fulton, 1950), and they were named Fulton’s strain (accession from FA-CMV to FE-CMV). Representatively, Yasuo classified CMV strains in tobacco into five strain-groups using 10 different host species (Yasuo, 1963; Li, 1995). Hollings et al. (1967) identified the CMV isolates from UK as PY/Y and II subgroups according to indicator plants. However, there are many strains of CMV. Kaper and Waterworth (1981) also list false identifications of new viruses that have turned out to be strains of CMV. Therefore, according to serology and phylogenetic analyses of *CP* genes, CMV isolates have recently been characterized into two subgroups, I and II. Nucleotide sequences of *CP* genes in CMV isolates within each subgroup can share up to 95% nucleotide sequence identity, whereas isolates in different subgroups share 69–77% sequence identity (Palukaitis and Garcíaarenal, 2003). Normally, high-temperature tolerance is greater in subgroup I than in subgroup II, while symptoms in subgroup II are less severe. Peppers are more likely to be infected with subgroup I isolates, since these are more prevalent in the warm climates where peppers are grown (Moury and Verdin, 2012). Subgroup I can be further divided into subgroups IA and IB, and populations of subgroup IB is very common in East Asia (Palukaitis et al., 1992; Roossinck et al., 1999).

Although accurate classification of serology and phylogenetic, the relationship between pathogenic genes in the virus and resistance genes in hosts was still unknown. Development a set of differential hosts of CMV strains have implications for breeding and reflect the relationship between resistance phenotypes and genotypes. Yang et al. (1992) identified a set of *C. annuum* lines with different levels of resistance as differential hosts for distinct CMV strains in pepper (Table 2). As a result, five strain-groups in pepper were identified in China, including CMV-P0, CMV-P1, CMV-P2, CMV-P3, CMV-P4, and CMV-P5. Moreover, several studies about strain specific resistance to CMV was also reported in pepper (Kang et al., 2010; Min et al., 2014; Choi et al., 2018).

**TABLE 2:** Differential hosts of CMV strains in pepper.

Name of strains	Resistant phenotype of differential hosts of *C. annuum*					Symptom of pepper
	LS-8501	LS-8502	LS-8503	LS-8504	LS-8505	
CMV-P0	R	R	R	T	S	Moderate mosaic
CMV-P1	R	T	T	S	S	Ring spot
CMV-P2	R	S	S	S	S	Ferny leaf to linear leaf
CMV-P3	T	S	S	S	S	Stem necrosis
CMV-P4	S	S	S	S	S	Yellow mosaic

*R, resistant; T, tolerance; S, susceptible.*

## Disease Screening Methods

Most purification methods for CMV strains used in practice are modifications of previously described methods (Scott, 1963; Lot et al., 1972). To prepare an inoculum that can infect pepper, purified CMV strains are propagated in leaves of tobacco (*Nicotiana benthamiana*). The virus inoculum is prepared by grinding 1 g of CMV-infected tobacco leaves in 5 ml of 0.03 M phosphate buffer (pH 7.2), containing 0.2% ascorbic acid or sodium diethyldithiocarbamate (Feng et al., 1986). The homogenate is filtered through gauze and mixed with 600-mesh carborundum as the inoculum.

The optimal time point for the identification of resistance to CMV in pepper is at an early stage of plant growth (Agrios et al., 1985; Dogimont et al., 1994). Generally, seedlings are inoculated with inoculum at the cotyledon stage to 6-leaf stage (cotyledon stage, 1–2 leaf stage, 3–4 leaf stage, and 5–6 leaf stage) by manual rubbing (Caranta et al., 1997; Chaim et al., 2001a; Yao, 2013; Min et al., 2014). According to Tian et al. (1989), the 3–6 leaf stage is the best inoculation period for assessing the resistance level of pepper resources because young seedlings can show a higher disease index at the cotyledon stage and 1–2 leaf stage, while old seedlings show lower values at the 7–8 leaf stage. Seedlings are rinsed 2 min after inoculation to remove excess inoculum (Dogimont et al., 1994). Following inoculation, the plans are maintained at 22–28°C under a 12-h day/12-h night cycle in a growth chamber (Feng et al., 1986; Dogimont et al., 1994).

Normally, the inoculated seedlings are observed for systematic symptom development after 2–4 weeks. Resistance is assessed according to the severity of mosaic and leaf distortion on a scale (Feng et al., 1986; Monma and Sakata, 1996; Chaim et al., 2001a; Zou, 2005; Yao et al., 2013; Rahman et al., 2016), number of local lesions (Caranta et al., 1997) or ELISA absorbance values (Reddy et al., 2001; Kang et al., 2010). The most commonly used method for disease evaluation is examining visually and assigning with a disease grade. Zou (2005) described the standard of disease grade, a scale ranging from 0 to 9, as following. 0 = no symptoms, 1 = mild mosaic on inoculated leaf, no leaf distortion, 3 = mild mosaic, stem streak and leaf distortion, 5 = strong mosaic, mild leaf distortion and stem necrotizing, 7 = severe mosaic and distortion, and 9 = severely stunted and systemic necrotizing. Disease indices of a population were calculated from disease grades of individual plants of that population, according to the formula disease indices (DIs) = (Σ (numbers of plants in the disease grade × disease grade)/9 × total numbers of plants) × 100 (Zou, 2005; Yao et al., 2013, Guo et al., 2017). Besides, Chaim et al. (2001a) rated the severity of the mosaic and leaf distortion of plants using a scale of 0–3, Monma and Sakata (1996) defined a score of 1 to 4. Although there are differences in the standard of disease grade, all of them can accurately reflect the resistance level of plants. Caranta et al. (1997) assessed the resistance and defined a disease score of 1–4 using the number of local lesions. “1” = 0 to 5 lesions, “2” = 6 to 20 lesions, “3” = 21 to 50 lesions, and “4” = more than 50 lesions, and resistance was assessed 4 days after inoculation (Caranta et al., 1997). However, the disease evaluation of number of local lesions depends on the CMV strain and mode of host resistance. The Fulton CMV/N strain could induce necrotic local lesions on the inoculated leaves, that gave an estimate of resistance by directly and quantitatively method of number of local lesions (Troutman and Fulton, 1958; Caranta et al., 1997). Kang et al. (2010) tested systemic infection using ELISA and monitored the symptoms of plants simultaneously. ELISA values could be more precise programs by providing a quantitative evaluation of viral resistance.

## Pepper Genetic Resources for CMV Resistance

The identification of sources of CMV resistance is a prerequisite for strategic breeding aimed at developing stable resistant varieties and hybrids. An accession of the wild pepper *C. baccatum*, “Pen 3-4,” was identified as resistant to CMV in greenhouse and field experiments (Pochard, 1977). Subsequently, various resistant or tolerant hybrids and breeding lines have been developed from the source, such as “Vania,” “Gadir,” “Pochard’s androgenic dihaploids” (ADH), and “Nicklow’s Emerald Bell” (Pochard and Daubèze, 1989; Caranta et al., 2002). The small-fruited Indian chili “Perennial,” which is the most well-characterized source of resistance to a broad range of viruses, shows tolerance or partial resistance to CMV (Pochard, 1977; Nono-Womdim et al., 1993; Lapidot et al., 1997). Another small-fruited pungent pepper accession, *C. frutescens* “BG2814-6,” possesses alleles conferring more complete resistance than that in Perennial (Grube et al., 2000). BG2814-6 crossed with Perennial has been used for the development of lines with improved CMV resistance by Mazourek et al. (2009). Suzuki et al. (2003) identified five resistant lines, “Sapporo-oonaga,” “Nanbu-oonaga,” “LS 1839-2-4,” “PI 439381-1-3,” and “Tabasco,” from 37 cultivated and wild *Capsicum* species. Previous study identified a CMV resistance source, Likeumjo, which was later used to develop commercial varieties, such as “Bukang” (Kang et al., 2010). Yao (2013) evaluated several breeding lines and accessions of *C. annuum* and found a resistant accession “BJ0747-1-3-1-1.” Screening of 199 genetic pepper resources by Shin et al. (2013) resulted in the identification of 32 resistant or moderately resistant *Capsicum* accessions to CMV_*P*__1_. Min et al. (2014) reported three accessions, “I7339,” “ICPN-18-8,” and “Daedlbo,” with resistance to CMV_*P*__1_. More recently, several accessions with resistance to CMV were identified from 50 pepper resources (Naresh et al., 2016). Rahman et al. (2016) identified two *C. annuum* genotypes, CA23 (Noakhali) and CA12 (Comilla-2) possess genes conferring resistance and moderate resistance phenotype to CMV, respectively. Choi et al. (2018) identified an Indian *C. annuum* cultivar, Lam32, with broad-spectrum resistance to CMV_*Korean*_, CMV_*FNY*_, and CMV_*P*__1_. In China, at least 317 sources of resistance or moderate resistance to CMV have been identified in *Capsicum* spp. by breeders in different groups, such as the Chinese local variety “erfutou,” “shifangjiao,” and “Hanchuan sweet pepper” (Li and He, 2000; Wang et al., 2001; Mao et al., 2004; Sun et al., 2008). Of note, the World Vegetable Center (AVRDC, Asian Vegetable Research and Development Center) has published a catalog of sources of resistance to viruses for pepper (*Capsicum* spp.), listing 165 *Capsicum* accessions with resistance and 46 accessions with tolerance to CMV (Green and Kim, 1994). These sources of resistance to CMV provide a practical reference and a basis for CMV resistant breeding (Table 3); however, most of the sources display only partial resistance to CMV, limiting resistance breeding in pepper.

**TABLE 3:** Primary genetic sources of resistance to CMV.

*Capsicum* species	Original source	Developed source	Resistance reaction	References
*C. annuum*	Perennial (IHR 384)	HAD 249, HAD 260, HAD 268, HAD 293	Resistance or tolerance to several CMV isolates	Singh and Thakur, 1977; Pochard et al., 1983
*C. annuum*	Wild type from Nicaragua	Rama	Resistance to CMV	Pochard, 1982
*C. annuum*	Milord		Partially resistant (reduced virus migration), resistance is suppressed at low temperatures (12–25^*o*^C)	Pochard and Daubèze, 1989; Nono-Womdim et al., 1991
*C. annuum*	Pusa Sadabahar, RHRC-clustering erect, Punjab Lal		Resistance to CMV	Pinaki, 1999
*C. annuum*	MRCH		Tolerance to CMV	Shifriss and Cohen, 1987
*C. annuum*	Likeumjo	Bukang	Resistance to CMV_*Korean*_ and CMV_*FNY*_ strains	Kang et al., 2010
*C. annuum*	Changyang difangzhong 4	BJ0747-1-3-1-1	Resistance to Hubei CMV isolates	Yao et al., 2013; Li N. et al., 2018
*C. annuum*	I7339, ICPN-18-8, Daedlbo, Lam3		Resistance to CMV_*P*__1_ strain	Min et al., 2014; Choi et al., 2018
*C. annuum*	CA23 (Noakhali), CA12 (Comilla-2)		Resistance to CMV subgroup-II isolate	Rahman et al., 2016
*C. baccatum*	pendulum 3-4 (Pen 3-4)	Vania, Philomèle 1	Resistance to CMV-N and CMV-MES, partial resistance Resistant to CMV migration	Pochard, 1977; Lapidot et al., 1997
*C. baccatum*	PI 439381-1-3		Resistance to CMV-Y	Suzuki et al., 2003
*C. frutescens*	LP-1		Resistance to CMV	Barrios et al., 1971
*C. frutescens*	BG2814-6		Resistance to several CMV strains	Grube et al., 2000
*C. frutescens*	PBC688		Resistance to CMV_*FNY*_	Guo et al., 2017
*C. frutescens*	LS 1839-2-4		Resistance to CMV-Y	Suzuki et al., 2003
*C. frutescens*	Tabasco		Resistance to CMV-Y	Suzuki et al., 2003

## Inheritance Studies and Genetic Mapping

The genetic basis of resistance to CMV in pepper is complex, and most studies have found partial resistance mediated by multiple loci (Grube et al., 2000; Suzuki et al., 2003; Yao et al., 2013; Wang, 2016). Table 4, displays classical genetic studies of resistance to CMV in different *Capsicum* accessions. Resistance in “Perennial” exhibited different inheritance patterns: monogenic recessive, partially dominant, or polygenic recessive (Singh and Thakur, 1977; Rusco and Csillery, 1980; Pochard and Daubèze, 1989; Bansal et al., 1992; Nono-Womdim et al., 1993; Lapidot et al., 1997; Wang, 2016). The line “Vania,” derived from *C. baccatum* “Pen 3-4,” showed dominant partial resistance to CMV (Caranta et al., 2002). The inheritance of CMV resistance in “BG2814-6” was incomplete and was controlled by at least two major recessive genes (Grube et al., 2000). The application of a mixed inheritance model revealed two additive-dominant major genes with additive-dominant polygenic traits in the CMV-resistance source “BJ0747-1-3-1-1” (Yao et al., 2013). Polygenic recessive resistance has been reported in “IHR 2451” and “IHR 4503” by Naresh et al. (2016). Single-gene control of resistance against CMV has also been reported. Kang et al. found that resistance in “Bukang” is under the control of a single dominant gene (Kang et al., 2010). Min et al. (2014) reported two different recessive genes conferring resistance to CMV in *C. annuum* “I7339.” More recently, Choi et al. (2018) showed that resistance in *C. annuum* “Lam32” is controlled by a single recessive gene, CMV resistance gene 2 (*cmr2*). Zhang et al. (2016) also described a single recessive gene *cr* underlying resistance to CMV.

**TABLE 4:** Classical genetic studies of resistance to CMV in *Capsicum.*

*Capsicum* species	Resistant parent	Genetic control and mechanisms of resistance	References
*C. annuum*	Perennial	Monogenic recessive, *cm*	Singh and Thakur, 1977
		Recessive genes	Pochard, 1982
		Polygenetic recessive inheritance, resistant to virus multiplication	Pochard and Daubèze, 1989
		Monogenic recessive and partially dominant	Rusco and Csillery, 1980
		Two or three incompletely dominant genes	Lapidot et al., 1997
		Two additive-dominant major genes with epistatic polygene or two additive-dominant major genes with additive-dominant polygene	Wang, 2016
*C. annuum*	Rama	A single dominant gene *Riv*	Pochard, 1982
*C. annuum*	MRCH	Polygenetic inheritance and linked to small fruit size	Shifriss and Cohen, 1987
*C. annuum*	Vania	Polygenetic inheritance, resistant to CMV migration	Caranta et al., 2002
*C. annuum*	Bukang	A single dominant gene *Cmr1*	Kang et al., 2010
*C. annuum*	BJ0747-1-3-1-1	Two additive-dominant major genes with additive-dominant polygene	Yao et al., 2013
*C. annuum*	I7339	Two recessive, *cmr3E* and *cmr3L*	Min et al., 2014
*C. annuum*	Q132	Monogenic recessive, *cr*	Zhang et al., 2016
*C. annuum*	Lam32	a single recessive gene *cmr2*	Choi et al., 2018
*C. frutescens*	LP-1	Monogenic recessive	Barrios et al., 1971
*C. frutescens*	BG2814-6	Incompletely dominant and at least two major recessive genes	Grube et al., 2000
*C. frutescens*	PBC688	Incompletely dominant and quantitatively inherited	Guo et al., 2017

Quantitative inheritance of resistance to CMV followed an additive-dominant genetic model, and the most important genetic effect was the additive one (Yan et al., 1996; Yu et al., 2000; Zou et al., 2004; Yao, 2013). High broad-sense heritability and narrow-sense heritability was detected for CMV resistance in different resistant lines (Yan et al., 1996; Zou et al., 2004; Yao, 2013; Herison et al., 2014). F_1_ plants showed intermediate in level of resistance, comparing to resistance and susceptible parents. Therefore, it is beneficial to obtain desirable resistance hybrid combinations if both parents have high resistance. However, breeding for CMV resistance totally based on phenotypic selection will take a longer time. The genetic mapping and marker-assisted breeding methods may accelerate the transfer of resistant traits.

The earliest QTL analysis of CMV-resistance in pepper was conducted by Caranta et al. (1997), that identified three resistance QTLs using a DH population of a cross between “Perennial” and “Yolo Wonder.” The three QTLs were located on chromosomes *Noir, Pourpre*, and linkage group 3, and together explained 57% of the total phenotypic variation. For each QTL, the Perennial allele increased the level of resistance. A digenic epistasis between one locus (TG66 on chromosome *Pourpre*) that controlled significant trait variation and another locus (TG124 on chromosome *Noir*) that by itself had no demonstrable effect on the trait was found to have an effect on CMV resistance (Caranta et al., 1997). Several other studies have identified and analyzed QTLs for CMV resistance in “Perennial“ (Chaim et al., 2001a; Zhao et al., 2011; Wang, 2016). Chaim et al. (2001a) identified four QTLs for CMV resistance on linkage groups 4, 6, 11, and 13 using “Maor (TMV-resistant line, *L*^1^/*L*^1^ genotype) × Perennial” populations. Whereas, one major QTL, *cmv11.1*, was detected in all three experiments and explained the higher percentage (16%–33%) of the observed phenotypic variation. This QTL is linked to the AFLP marker E35/M48–101 and *L* locus which confers resistance to tobacco mosaic virus (TMV). A linkage between resistance to CMV and susceptibility to TMV was observed in “Perennial.” Moreover, interaction that involved E35/M48–101, *cmv11.1*, and the RFLP marker TG191 on LG2 was detected. Therefore, choosing “Maor” allele at TG191 with “Perennial” allele at E35/M48–101 in “Perennial” could have a good level of resistance to CMV in pepper breeding. Markers linked to QTL for CMV resistance were also linked to QTL for fruit weight, as observed by Chaim et al. (2001b). This study also explained the cause of the relatively slow progress in the development of CMV-tolerant large-fruited pepper genotypes (Lapidot et al., 1997; Chaim et al., 2001a). The linked molecular markers facilitate the identification of genotypes from a breeding perspective. QTLs for CMV resistance were also detected on linkage groups 1, 4, and 7 by Zhao et al. (2011). Wang (2016) identified four QTLs for CMV resistance in “Perennial” using F_2_ (Carolina Wonder × Perennial) and RIL (83-58 × Perennial) populations. *CMV12.3*, which was detected in the RIL population, was also detected in the F_2_ population.

QTLs for CMV resistance have also been studied in other sources (Caranta et al., 2002; Yao et al., 2013; Min et al., 2016; Guo et al., 2017). Caranta et al. (2002) identified a QTL on chromosome 12 of *C. annuum* line “Vania” explaining 45–63.6% of the phenotypic variation, in addition to three minor QTLs on chromosomes 5 and 11. In the inbred line “BJ0747-1-3-1-1,” six QTLs, including one major effect QTL on chromosome 11 and several minor effect QTLs, conferred CMV resistance (Yao et al., 2013). A major QTL, explaining about 20% of the phenotypic variation on chromosome 11, was identified for resistance to CMV in “BJ0747-1-3-1-1” (Li N. et al., 2018). Two QTLs were detected on chromosomes 5 and 10 for CMV resistance in “A1” by Min et al. (2016). In a recent study by Guo et al. (2017), two QTLs on chromosomes 2 and 11 were shown to be associated with CMV resistance in “PBC688” in *C. frutescens*. *qCmr2.1*, on chromosome 2, was in the region containing *CA02g19570* and *CA02g19600*. *CA02g19570* exhibited high levels of expression after inoculation and is homologous to genes encoding N-like proteins associated with TMV resistance in *Solanum* crops.

A few monogenic, strain-specific resistance loci have been mapped (Kang et al., 2010; Park et al., 2011; Min et al., 2014). The first single dominance resistance gene, *Cmr1*, conferring resistance to CMV_*Korean*_ and CMV_*FNY*_ strains, was detected in *C. annuum* “Bukang.” *Cmr1* is in the region on chromosome 2 associated with the ToMV resistance locus *Tm-1* in tomato (Kang et al., 2010). The single recessive gene *cmr2*, resistant to CMV_*P*__1_, has been mapped to marker Affy4 (within 2.3 cM) on chromosome 8 (Choi et al., 2018). Two recessive genes, *cmr3E* and *cmr3L*, were located on linkage group 4, associated with the RAPD primer OPAT16 (Min et al., 2014). Zhang et al. (2016) mapped a recessive gene, *cr*, to a linkage group including two SSR markers GI1354 and genSSR3000.

## Possible Mechanisms of Host Resistance

Various mechanisms underlying resistance to CMV have been demonstrated, including the restriction of virus entry and uncoating (installation) in host cells (Lecoq et al., 1982; Caranta et al., 1997), restriction of viral multiplication (Nono-Womdim et al., 1993), or restriction of the long-distance movement of the virus (Dufour et al., 1989; Nono-Womdim et al., 1993). Different resistance mechanisms may be related to environmental conditions, CMV isolates, or genetic backgrounds (Chaim et al., 2001a). CMV resistance in *C. annuum* “Vania,” “Milord,” “L57,” and “L113” is governed at the level of long-distance movement and is classified as partial resistance (Caranta et al., 2002; Mihailova et al., 2013). The Indian chili “Perennial,” which is described as tolerant or partially resistant to CMV, shows the restriction of CMV installation, reduced viral multiplication, and the prevention of long distance movement (Nono-Womdim et al., 1993; Caranta et al., 1997). *C. frutescence* “BG2814-6” blocks viral replication and cell-to-cell movement (Grube et al., 2000). *C. annuum* “Bukang” inhibits the movement of CMV from the epidermal cell layer to mesophyll cells (Kang et al., 2010).

Cucumber mosaic virus infection affects defense-related enzyme metabolism in pepper plants, causing the accumulation of superoxide dismutase (SOD), peroxidase (POD), and polyphenol oxidase (PPO) activity, decreases in phenylalanine ammonia lyase (PAL) activity, and increases in the levels of lignin and total phenols. A correlation analysis has indicated that POD, PAL, PPO, lignin, and total phenols are correlated with resistance after inoculation, where resistant sources show high POD and PPO activity (Yang et al., 2006; Zhang, 2009).

In transcript profiles, several key genes associated with plant-pathogen interactions show increased transcript abundances after inoculation, including genes encoding chitinase, pathogenesis-related (PR) protein, TMV resistance protein, WRKY transcription factor, and jasmonate ZIM-domain protein (Zhu et al., 2018). Among them, four WRKY transcription factors, WRKY 6, WRKY 33, WRKY 38, and WRKY 45, responded to CMV infection in pepper. One possible candidate gene, *CA02g19570*, that control of CMV resistance in *C. frutescens* have been identified (Guo et al., 2017). Recent studies determined that *CA02g19570* was the gene of *qCmr2.1* governing CMV resistance in “PBC688” (Zhang et al., 2020). The expression of *CA02g19570* was significantly up-regulated after inoculation, with higher expression levels in the CMV-resistant parent “PBC688.” Silencing of *CA02g19570* gene exhibited severe systemic mosaic symptoms after inoculation with CMV (Zhang et al., 2020).

## Breeding Strategies for CMV Resistance

### Genetic Breeding for CMV Resistance

**FIGURE 3:**
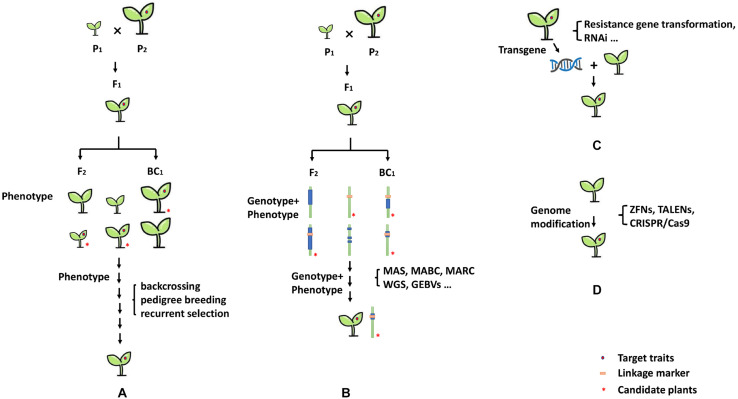
Breeding strategies for CMV resistance. **(A)** Genetic breeding strategies, **(B)** marker-assisted breeding and genome assistance breeding strategies, **(C)** transgenic approaches and RNAi strategies, and **(D)** genome modification strategy.

Traditional protocols for plant genetics and breeding, such as pedigree, backcrossing, and recurrent selection methods, have been applied to develop several breeding lines and cultivars with measurable levels of resistance (Figure 3A). For example, the bell-pepper inbred line “Vania” with CMV-resistant trait was developed from recurrent selection using *C. baccatum* “Pen 3-4” as a primary genitor and *C. annuum* “Antibois” and “Bastidon” as secondary genitors for resistance to CMV (Pochard and Daubèze, 1989). The inbred line “Vania” cumulated different CMV resistance genotypes and carried wide-range genetic diversity at the same time. Another example was the big-fruit sweet pepper breeding inbred line “98-42,” which was developed by Guo with CMV resistance and is widely used in China. Using the breeding line “98-42,” a series of commercial varieties, including “Zhongjiao No.4,” “Zhongjiao No.6,” and “Zhongjiao No.7,” were developed by IVF, CAAS (Institute of vegetables and Flowers, Chinese Academy of Agricultural Sciences), with good fruit yield and tolerance to CMV (Guo, 1994; Yu et al., 2019). Additionally, strategy can be elaborated doubled haploid (DH) methodology for speeding up the process of breeding lines against CMV and obtaining completely homozygous pure resistant lines. The successful applications of DH strategy for pepper resistant breeding have been described by several seed companies.

### Marker-Assisted Breeding for CMV Resistance

Conventional breeding involves hybridization with contrasting parental lines and the selection and evaluation of individual plants in cross and backcross populations. However, it is often very difficult and time-consuming to evaluate phenotype or pathotype resistance. The application of marker-assisted selection (MAS) has facilitated breeding for crop improvement, especially for phenotype traits that controlled by quantitative trait loci or recessive allele. Additionally, it is useful for backcross breeding, for the introgression of resistance genes from wild species while selecting against the undesirable characteristics of the wild parent (Young and Tanksley, 1989). Marker-assisted selection has been successfully used in efficient selection of many resistance genes in pepper crop improvement (Ridzuan et al., 2018). Alternatively, several modern breeding strategies, such as, marker-assisted backcrossing (MABC), marker-assisted recurrent selection (MARS), and marker-assisted pedigree selection (MAPS), have been also used for resistant breeding in pepper. These methods are essentially one type of MAS, all of which can quickly and efficiently introduce target genes while retaining the essential characteristics of the recurrent parent (Figure 3B). For CMV-resistance breeding, CMV-susceptible and -resistant genotypes can be precisely identified using molecular markers at an early stage of plant growth, without requiring field screening with artificial inoculation or any environmental influence. Reported linkage markers associated with CMV-resistant traits in *Capsicum* are listed in Table 5, and the utilization of the linkage markers in MAS breeding program for CMV-resistant has been reported in pepper (Zhang et al., 2016; Kim et al., 2017).

**TABLE 5:** Linkage markers associated with CMV-resistant in *Capsicum*.

Genetic population	Resistant loci	Chromosome/Linkage group	Marker type	Marker name	Marker size (bp)	Map location (cM)	CMV strains	References
Maor × Perennial	*cmv11.1*	LG11/Chr. 11	AFLP	E35/M48-101	101	–	CMV-V27	Chaim et al., 2001a
	*cmv13.1*	LG13	RAPD	OPP11_500_	500	–		
	*cmv6.1*	LG6	RAPD	OPA12_1700_	1700	–		
	*cmv4.1*	LG4	AFLP	E48/M49-270	270	–		
H3 × Vania	*cmv12.1*	Chr. 12	AFLP	E33/M48-132	132	–	CMV-MES and CMV-N	Caranta et al., 2002
	*cmv11.1*	Chr. 11		L	–	–	CMV-N	
	*cmv11.2*	Chr. 11		P14/M59-137	137		CMV-N	
	*cmv5.1*	Chr. 5		E35/M48-475	475		CMV-N	
VC16a × SS69	-	–	ISSR	I-34450	450	27.3	-	Wang et al., 2009
Bukang × Jeju	*Cmr1*	Chr.2	SNP	CaTm-int3HRM	158	2	CMV_*Korean*_, CMV_*FNY*_ and CMV_*P*__1_	Kang et al., 2010
				CaT1616BAC	200	2		
				240H02sp6	472	2		
BJ0747-1-3-1-1 × XJ0630-2-1-2-1-1	*qcmv.hb-4.1*	LG4	ISSR	UBC843		–	CMV-HB	Yao et al., 2013
	*qcmv.hb-8.2*	LG8	ISSR	UBC829		–		
BJ0747-1-3-1-1 × XJ0630-2-1-2-1-1	*qcmv11.1*	Chr. 11	SNP	Marker6201026	–	–	CMV_*HB–zj*_	Li N. et al., 2018
	*qcmv11.2*	Chr. 11		Marker5409028	–	–		
	*qcmv12.1*	Chr. 12		Marker17652010	–	–		
Q132 × FS871	*cr*	–	SSR	GI1354, genSSR3000	– –	1.3 0.3	–	Zhang et al., 2016
Perennial × 83-58	*CMV12.3*	Chr. 12	KASP	UN54228_1146	–	1.8	–	Wang, 2016
PBC688 × G29	*qCmv2.1*	Chr. 2	Indel	Indel2-134	230	0.3	CMV_*FNY*_	Guo et al., 2017
I7339 × E1338	*cmr3E, cmr3L*	–	RAPD	OPAT-16		22.3 20.7	CMV_*P*__1_	Min et al., 2014
A1 × 2602	*cmvP1-5.1*	Chr. 5	SNP	SNP13525, SNP13624	–	–	CMV_*P*__1_	Min et al., 2016
	*cmvP1-10.1*	Chr. 10	SNP	SNP07415, SNP07478	–	–		
Lam32 × Jeju	*cmr2*	Chr. 8	KASP	Affy4	–	2.3	CMV_*Korean*_ and CMV_*FNY*_	Choi et al., 2018

*Chr., chromosome; LG, linkage group; AFLP, amplified fragment length polymorphism; RAPD, random amplified polymorphic DNA; ISSR, inter-simple sequence repeat; SNP, single nucleotide polymorphism; CAPS, cleaved amplified polymorphic sequence; SSR, simple sequence repeat; KASP, kompetitive allele-specific PCR; Indel, insertion-deletion.*

### Genome Assistance Breeding for CMV Resistance

The technological advancement next-generation sequencing (NGS) and single nucleotide polymorphism (SNP) genotyping, provides powerful tools for crop improvement (Figure 3B). High-throughput SNP genotyping offers a number of advantages over previous marker systems, such as an abundance of markers, rapid processing of large populations, and low cost (Thomson, 2014). It is particularly useful for breeders to use SNP-based targeted approach to select and combine beneficial alleles at known major genes controlling traits of interest in crop breeding. Moreover, high-throughput SNP genotyping also provides the genome-wide selection (GWS) approaches based on genomic estimated breeding values (GEBVs), rather than being limited to a few known loci. Several high-throughput SNP genotyping platforms, including Affymetrix Axiom array, Fluidigm dynamic arrays, restriction-enzyme-based genotyping-by-sequencing (GBS), and Illumina Infinium iSelect HD array have been reported (Thomson, 2014). Recently, Kim et al. (2017) reported successfully development of Fluidigm SNP type genotyping assays for marker-assisted selection breeding in pepper, that could simultaneously analyze 20 SNP markers about target breeding traits. It will provide an efficient application for MAS (also including MABC, MARS, and MAPS, etc.), and easily tracks with flanking SNP markers polymorphic between the donor and recurrent parent.

### Transgenic Approaches for Breeding Against CMV

The use of genetic transformation of pepper with CMV resistance gene is a strategy to obtain resistant plants, such as application of *CA02g19570* of *qCmr2.1* loci. Another successfully strategy against CMV infection in pepper crops is RNA-mediated virus gene silencing (Figure 3C). RNA interference (RNAi) or RNA silencing, which was first discovered in plants, is a natural defense mechanism against viruses (Hamilton and Baulcombe, 1999). It is triggered by the processing of virus-derived dsRNA by host Dicer-like (DCL) enzymes into 21- to 24-nucleotide (nt) small interfering RNAs (siRNAs) (Blevins et al., 2006; Fusaro et al., 2006; Pooggin, 2018). The expression of dsRNA from transgene plants can induce post-transcriptional gene silencing (PTGS) and transcriptional gene silencing (TGS). Plants expressing a copy of a viral gene in sense and/or antisense orientation have shown resistance upon infection with the virus (or other virus containing identical sequences) through RNAi (Waterhouse and Helliwell, 2003). Therefore, RNAi can be used to engineer resistance in plants against viruses. The transgenic pepper plants that express CMV-siRNA displayed delayed symptom development and significantly milder disease severity upon inoculation with CMV strains (Kim et al., 1997; Cai et al., 2003; Lee et al., 2009). In addition, it is a potential strategy to use transgenic plants for introduced defense-related genes, which are involved in the JA signaling and antiviral RNA silencing pathways that regulate against virus resistance (Yang et al., 2020).

### Genome Modification for Breeding Against CMV

In the past decade, great advancements in genome or gene editing technologies, such as zinc finger nucleases (ZFNs), transcriptional activator-like effector nucleases (TALENs), and clustered regularly interspaced short palindromic repeats associated to nuclease Cas9 (CRISPR/Cas9)., has made it possible to precisely target any gene of interest (Jaganathan et al., 2018; Van Eck, 2018; Xu et al., 2019). Because of simplicity, precision and power, genome editing offers great opportunities to develop improved crop varieties with clear-cut addition of valuable traits or removal of undesirable traits. Moreover, gene editing is more precise than either conventional crop breeding methods or standard genetic engineering methods (Figure 3D). To date, these technologies are being applied in many plant species for enhancing qualities, yield, disease resistance, and other traits (Ito et al., 2015; Pyott et al., 2016; Li R. et al., 2018). Several cloned-genes conferring resistance to virus diseases have sequence variation between resistant and susceptible loci (Li et al., 2016), which might provide good sites for genome modification. Therefore, modification of the sequence of susceptible loci using genome-editing strategy has the potential to improve pepper varieties against CMV.

## Conclusion and Future Prospects

Cucumber mosaic virus is one of the most serious threats to pepper production worldwide. Hence, the utilization of resistance resources for breeding resistant cultivars is an efficient method to control CMV outbreaks. The identification and characterization and introgression of new CMV resistance sources and QTLs in pepper within the previous studies have significantly contributed to the effective control of the disease. However, many CMV resistance-related issues still need to be unraveled. The levels of CMV resistance in known disease resistance resources are still insufficient and mostly partial resistance. Hence, it is requiring us to screen new and more desirable sources to diversify the genetic basis of the resistance. Recently several new sources with good levels of CMV resistance have been identified within *C. annuum*, which have been utilized for developing peppers with improved resistance (Rahman et al., 2016; Yu et al., 2019). It is suggesting the possibility of developing commercially accepted cultivars with broad spectrum and durable resistance. The development of markers linked to resistance loci is the most convenient means for CMV resistance breeding. Advances in NGS technologies, genome-wide association studies (GWAS), high-throughput SNP genotyping and fine mapping of resistance genes or QTLs facilities the development of reliable markers, map-based cloning and accurate transfer to elite lines via MAS or GWS in pepper. The genetic basis of resistance to CMV is quite complex, involving multiple genes, recessive or partially dominant. The mechanism of resistance to CMV in pepper still stays at observing the virus migration, viral multiplication and defense-related enzyme metabolism. There is still a lack of understanding of the resistance mechanisms at the molecular level. Thus, it is necessary to deep the study of the interaction between virus and host, clarifying the host gene-mediated virus resistance mechanisms and signaling in pepper. However, molecular and genetic studies are difficult in pepper, which hinders the identification of genes responsible for CMV resistance. So far, only *CA02g19570* that control of CMV resistance in *Capsicum* was cloned, more resistance genes need to be identified. Remarkable technological achievements in the field of DNA sequencing will be important for identifying genes associated with CMV resistant traits. Genome editing provides great promise to improve crop productivity but relies on genetic transformation and plant regeneration, which are bottlenecks in the process (Altpeter et al., 2016; Xu et al., 2019). However, efficient protocol for transformation and regeneration from tissue culture remain arduous for pepper, successful genetic transformation of pepper is strongly dependent on the genotype. The transformation and regeneration system should be further optimized in pepper. Recently, Maher et al. (2020) described new methods to generate gene-edited dicotyledonous plants through *de novo* meristem induction, that could overcome the difficult of transformation and regeneration in plant gene editing.

Summarize, future efforts to identify new and more desirable sources of resistance and new resistance genes and QTLs are imperative. With the advent of novel technologies, precise markers and approaches can facilitate the progress for resistant breeding. Preliminary results from these studies have been encouraging, suggesting the possibility of developing commercially accepted cultivars with good levels of CMV resistance.

## Author Contributions

NL and CY: writing and editing. YY and SG: literature collection. FW: project administration. CJ and MY: funding acquisition. All authors have read and agreed to the published version of the manuscript.

## Conflict of Interest

The authors declare that the research was conducted in the absence of any commercial or financial relationships that could be construed as a potential conflict of interest.

## Footnotes

1 www.fao.org
